# Supplementary material for: Aneuploidy as a cause of impaired chromatin silencing and mating-type specification in budding yeast
Source: eLife. 2017 Aug 25;6:e27991. doi: 10.7554/eLife.27991 (PMC5779231; doi:10.7554/eLife.27991)
Supplement: Supplementary file 3. — Genes leading to the loss of HML::YFP silencing when copy number is increased are listed with a functional description and a desilencing score. The desilencing score was calculated as the average YFP intensity in WT haploid strains carrying individual candidate genes on a low-copy (centromeric) plasmid, relative to the average YFP fluorescence in the disome X strain. Average YFP intensities were calculated using three biological replicates per strain. [file elife-27991-supp3.docx]

| **Table 3.** List of top 15 candidates from gain-of-copy-number screen. | | |
| --- | --- | --- |
| **Genotype** | **Desilencing score relative to disome X** | **Description of gene** |
| Disome X | 1 | -- |
| *WT+SRS2* | 0.568966 | DNA helicase and DNA-dependent ATPase; involved in DNA repair and checkpoint recovery, affects genome stability; functional homolog of human RTEL1. |
| *WT+DPB11* | 0.53 | DNA replication initiation protein; loads DNA pol epsilon onto pre-replication complexes at origins; ortholog of human TopBP1. |
| *WT+RPL39* | 0.489655 | Ribosomal 60S subunit protein L39; required for ribosome biogenesis; loss of both Rpl31p and Rpl39p confers lethality; homologous to mammalian ribosomal protein L39. |
| *WT+ASF1* | 0.489483 | Nucleosome assembly factor; involved in chromatin assembly, disassembly. |
| *WT+RPS14B* | 0.481724 | Protein component of the small (40S) ribosomal subunit; required for ribosome assembly and 20S pre-rRNA processing; homologous to mammalian ribosomal protein S14 and bacterial S11. |
| *WT+PRE3* | 0.422759 | Beta 1 subunit of the 20S proteasome; responsible for cleavage after acidic residues in peptides. |
| *WT+SPT10* | 0.387069 | Histone H3 acetylase with a role in transcriptional regulation; involved in S phase-specific acetylation of H3K56 at histone promoters, which is required for recruitment of SWI/SNF nucleosome remodeling complex. |
| *WT+CYR1* | 0.358621 | Adenylate cyclase; the cAMP pathway controls a variety of cellular processes, including metabolism, cell cycle, stress response, stationary phase, and sporulation. |
| *WT+HIR3* | 0.331552 | Subunit of the HIR complex; a nucleosome assembly complex involved in regulation of histone gene transcription; involved in position-dependent gene silencing and nucleosome reassembly; ortholog of human CABIN1 protein. |
| *WT+RTT101* | 0.31569 | Cullin subunit of a Roc1p-dependent E3 ubiquitin ligase complex; role in anaphase progression; required for recovery after DSB repair. |
| *WT+RPA34* | 0.295 | RNA polymerase I subunit A34.5; essential for nucleolar assembly and for high polymerase loading rate; nucleolar localization depends on Rpa49p. |
| *WT+SWI3* | 0.291552 | Subunit of the SWI/SNF chromatin remodeling complex; SWI/SNF regulates transcription by remodeling chromosomes. |
| *WT+MIR1* | 0.269138 | Mitochondrial phosphate carrier; imports inorganic phosphate into mitochondria. |
| *WT+VPS25* | 0.268103 | Component of the ESCRT-II complex; ESCRT-II is involved in ubiquitin-dependent sorting of proteins into the endosome. |
| *WT+NUP85* | 0.254483 | Subunit of the Nup84p subcomplex of the nuclear pore complex (NPC); plays roles in several processes that may require localization of genes or chromosomes at the nuclear periphery, and chromatin silencing; homologous to human NUP85 aka NUP75. |
